# Mechanisms of Phthalate-Induced Accelerated Ovarian Aging in Experimental Models

**DOI:** 10.1007/s40572-026-00521-1

**Published:** 2026-02-13

**Authors:** Adira M. Safar, Jodi A. Flaws

**Affiliations:** Department of Comparative Biosciences, University of Illinois Urbana-Champaign, 2001 S. Lincoln Ave, Urbana, IL 61802 USA

**Keywords:** Phthalates, Ovarian aging, Female reproductive toxicity

## Abstract

**Purpose of Review:**

Accelerated ovarian aging is associated with early infertility as well as other adverse health outcomes. Little is known about the factors that accelerate ovarian aging, but several studies indicate that exposure to phthalates accelerates ovarian aging. This is a significant human health concern because humans are ubiquitously and unavoidably exposed to phthalates. Thus, it is imperative to study the mechanisms of phthalate-induced accelerated ovarian aging so that strategies can be developed to prevent phthalate-induced ovarian aging. This review focuses on the mechanisms by which phthalates cause ovarian aging in non-human experimental models and highlights gaps in the literature.

**Recent Findings:**

Phthalate exposure may accelerate ovarian aging and in turn, accelerate female reproductive aging through several mechanisms. Specifically, phthalates can alter steroidogenesis and folliculogenesis, ultimately dysregulating estrous cyclicity and decreasing fertility. Phthalate-induced disruptions in the brain and gut contribute to these changes. Additionally, phthalate exposures increase ovarian inflammation and oxidative stress, which contribute to accelerated ovarian aging. Phthalate exposure also increases ovarian autophagy, mitochondrial dysfunction, and apoptosis, which ultimately increase follicular atresia and accelerate depletion of the follicle reserve.

**Summary:**

Phthalates accelerate ovarian aging through numerous interlinked mechanisms that may be used as targets for prevention, inhibition, or reversal of phthalate-induced ovarian aging in patients experiencing infertility. Further studies should investigate the effects of environmentally relevant phthalate exposures on these mechanisms and explore therapies that target these mechanisms.

## Introduction

The female reproductive system is a sensitive indicator of aging, as it ages before most other physiological systems [1]. In females, early reproductive aging is associated with early onset of infertility as well as increased risk of heart disease, depression, osteoporosis, and even early death [1]. Reproductive aging is controlled, in part, by the ovary, as the ovary is responsible for follicular development and sex steroid hormone synthesis, and loss of ovarian follicles and declining sex steroid hormones are hallmarks of ovarian aging [1, 2].

Endocrine-disrupting chemicals such as phthalates target the ovary [2]. Phthalates are ubiquitous in the environment, as they are present in food packaging, medical tubing, children’s toys, and personal care products (Table 1) [2]. Phthalates are not covalently bound to the products that they are used in, so they tend to leach out of products at high temperatures, resulting in high levels of phthalate exposure via ingestion of contaminated foods and beverages [2, 3]. Phthalate exposure can also occur through inhalation and dermal absorption [2].

**Table 1:**
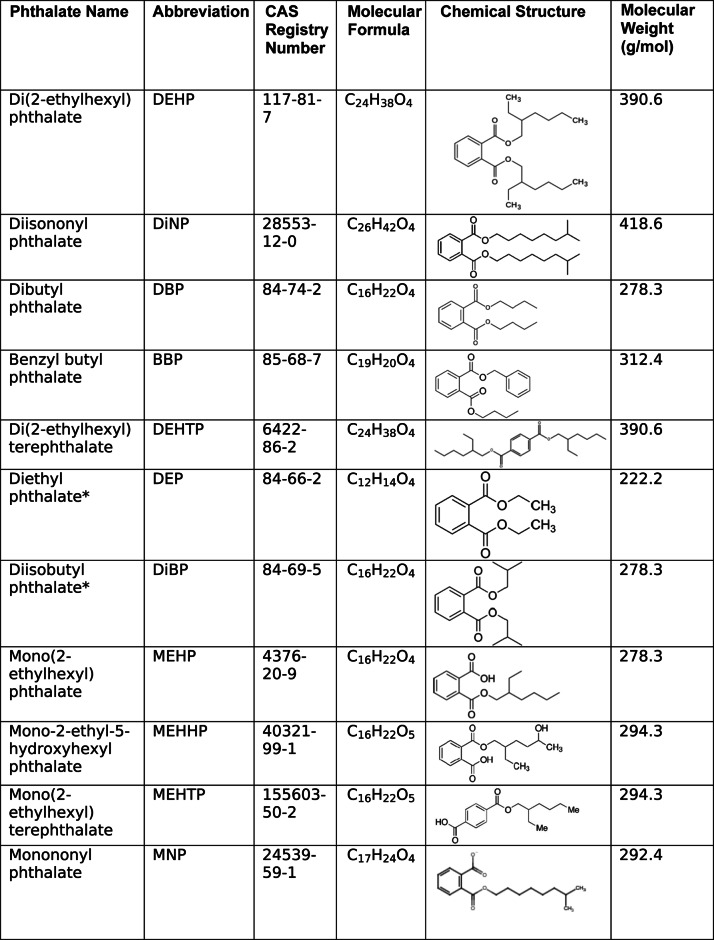

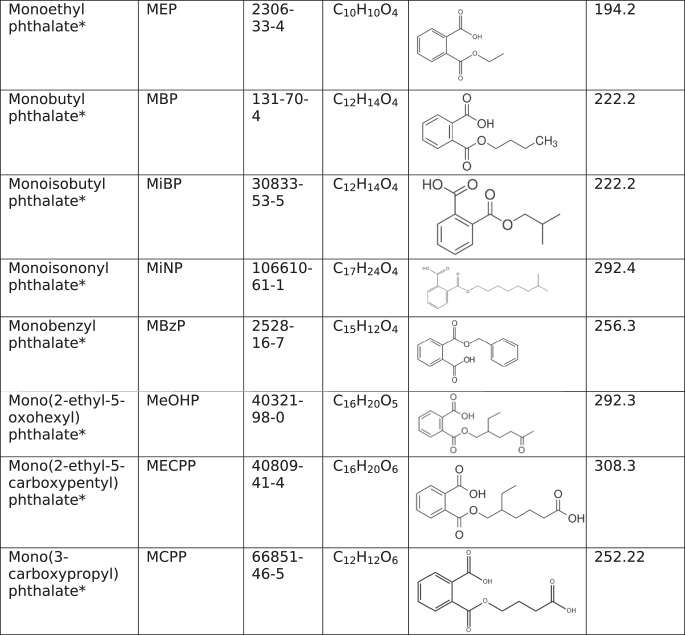
Properties of phthalates and phthalate metabolites

An asterisk (*) indicates that the phthalate or phthalate metabolite was reviewed only as a component of a mixture due to lack of research on the individual chemicals

Some studies support that phthalate exposures accelerate ovarian aging [4–8]. Due to their ubiquitous nature in the environment, phthalate exposure is unavoidable. While policy changes may reduce the use of specific phthalates in industry, an abundance of replacement chemicals exists that has not been tested for toxicity. Even in the absence or reduction of phthalate use in manufacturing, phthalate exposures would be markedly decreased, but not eliminated due to plastic pollution and widespread environmental contamination [9]. Additionally, the effects of phthalate exposures can last far beyond the initial window of exposure, so cessation of phthalate exposure would not eradicate phthalate-induced ovarian aging in the population that has already been exposed to phthalates [10, 11]. Thus, it is important to study the mechanisms of phthalate-induced ovarian aging so that therapies can be designed to mitigate or reverse phthalate-induced ovarian dysfunction in females. This article describes the systemic, ovarian, cellular, and molecular changes associated with phthalate-induced accelerated ovarian aging in non-human experimental models (Table 2).

**Table 2:** Summary of mechanisms thought to underlie phthalate-induced ovarian aging

Phenotypes of Phthalate-Induced Ovarian Aging Observed in Animal Models	Systemic Mechanisms	Ovarian Mechanisms	Cellular Mechanisms in the Ovary	Molecular Mechanisms in the Ovary
Accelerated the depletion of ovarian follicle pool	- HPO dysregulation - Gut bacterial dysbiosis induced LPS signaling to the ovary from the gut	- Increased follicular atresia	- Increased apoptosis - Excessive autophagy - Ferroptosis - Mitochondrial dysfunction	- Oxidative stress - PPARα/PPARγ activation - Reductions in IGF-1 pathway - Increased DDIT4 (induced excessive autophagy) - Decreased COX-2 (increased apoptosis)
Decreased estradiol and progesterone levels	- HPO dysregulation - Gut bacterial dysbiosis altered GUS enzyme activity in the gut (estradiol only)	- Accelerated depletion of ovarian follicle pool	- Mitochondrial dysfunction	- Altered steroidogenic enzyme expression - Oxidative stress - PPARα/PPARγ activation
Increased FSH levels and altered LH levels	- HPO dysregulation - Gut bacterial dysbiosis may induce LPS signaling to the hypothalamus and/or pituitary from the gut	- Decreased estradiol and progesterone production and/or secretion	N/A	N/A
Decreased fertility	- HPO dysregulation - Estrous cycle irregularity	- Accelerated depletion of ovarian follicle pool - Decreased estradiol and progesterone production and/or secretion - Anovulation - Fibrosis of the ovarian stroma	N/A	N/A
Estrous cycle irregularity	- HPO dysregulation	- Decreased estradiol and progesterone production and/or secretion - Anovulation	N/A	N/A
Fibrosis of the ovarian stroma	- Gut bacterial dysbiosis induced LPS signaling to the ovary from the gut	- Accelerated depletion of ovarian follicle pool	N/A	- Altered expression of ECM modulators - Oxidative stress - PPARα/PPARγ activation - Inflammation - Increased TNFα - NLRP3 activation - LPS signaling

N/A = not available

### Ovarian Aging

In humans, physiological ovarian aging is characterized by the loss of oocyte quality and quantity through depletion of the ovarian follicle pool [12]. Females are born with a finite number of primordial follicles that are gradually depleted throughout the reproductive lifespan, and the rate of follicular decline accelerates with increased age [12, 13]. Declines in the production of sex steroid hormones such as estradiol and progesterone accompany the natural loss of ovarian follicles, and reductions in sex steroid hormones lead to dysregulation of the hypothalamic-pituitary-ovarian (HPO) axis, resulting in fluctuations in menstrual cyclicity and loss of fertility [13]. The reproductive lifespan is terminated at menopause, which is characterized by the cessation of menstrual periods and marks the end of ovarian function and fertility [13, 14].

Some rodents are models of human physiological ovarian aging. Physiological ovarian aging in rodents is characterized by loss of the ovarian follicle reserve with each cycle, which accelerates with increased age, resulting in loss of fertility [14, 15]. Similar to humans, mice exhibit loss of ovarian follicles and declining estradiol and progesterone levels with age, which causes HPO dysregulation [15]. Further, rodents experience increasing estrous cycle irregularity accompanied by decreasing fertility due to oocyte depletion [14]. Mice can also enter a persistent anovulatory state known as estropause [14]. C. elegans and Drosophila also serve as basic models for reproductive and ovarian aging in humans, as they experience decreased reproductive potential with increased age [14].

### Accelerated Ovarian Aging

Menopause typically occurs in humans around age 51; however, some females experience menopause earlier than others [13]. Premature ovarian insufficiency (POI) is a condition that occurs when a female enters menopause before age 40 [14]. POI affects around 1% of females; however, the causes of 90% of POI cases are unknown [13, 14]. Further, prematurely low estradiol levels in POI patients pose more serious health consequences than those that experience menopause around age 50–51 [14]. Such health consequences include increased mortality and higher prevalence of cardiovascular disease, autoimmune conditions, osteoporosis, cognitive dysfunction, mood disorders, and sexual disorders [14].

POI and early menopause are characterized by premature follicle loss, reduced estradiol levels, increased follicle-stimulating hormone (FSH) levels, and premature menstrual irregularities and infertility [13, 14]. Recent literature demonstrates that environmental toxicant exposure can accelerate ovarian aging in humans, resulting in ovarian and reproductive pathologies [13]. Interestingly, numerous studies also suggest a link between gut bacterial dysbiosis and accelerated ovarian aging in both humans and mice [16].

Animal models can be used to model accelerated ovarian aging. While rodents do not naturally experience accelerated ovarian aging, phenotypes of accelerated ovarian aging have previously been induced in rodent models under various conditions, including in response to phthalate exposures [1, 7, 8, 13, 17–19]. Rodents are excellent models for studying mechanisms of ovarian aging, as they share a considerable amount of their reproductive physiology with humans [15]. Quail, zebrafish, and rotifers can also be used as models for human ovarian aging at the molecular level, though to a lesser extent, due to their shared gene homology and hormonal control of reproduction with humans [20–22].

### Mechanisms of Ovarian Aging

Current mechanisms understood to be involved in ovarian aging in humans include HPO dysregulation, mitochondrial dysfunction, oxidative stress, DNA damage, alterations in apoptosis and autophagy, fibrosis of the ovarian stroma, chronic inflammation, telomere shortening, alterations in protein homeostasis, epigenetic alterations, vascular aging, and alterations in meiosis resulting in aneuploidy; however, the mechanisms of phthalate-induced ovarian aging remain unknown [12, 23, 24]. Interestingly, numerous studies suggest a link between phthalate exposures and ovarian aging through induction of some of these mechanisms in non-human experimental models [2, 4, 5, 11, 25–48].

### Phthalate-Induced Accelerated Ovarian Aging

Phthalate exposure is associated with POI, indicating that phthalate exposure may accelerate ovarian aging in humans [13]. Phthalate exposures play a complicated role in ovarian aging and decrease fertility in several animal models [5, 6, 11, 15, 27, 36–38]. In rodents, phthalate exposures can dysregulate estrous cyclicity and alter ovarian steroidogenesis and folliculogenesis in a manner consistent with accelerated ovarian aging [2, 4, 11, 26, 32, 33, 37–50]. Interestingly, the brain, gut microbiome, and ovary communicate through FSH, luteinizing hormone (LH), and lipopolysaccharide (LPS) signaling, and this communication plays a role in inducing the observed ovarian aging phenotypes in response to phthalate exposures [2, 4, 37, 41].

Several mechanisms are thought to underlie the phthalate-induced accelerated depletion of the follicle pool observed in rodents. Specifically, phthalate exposures increase follicular atresia through increased apoptosis, mitochondrial dysfunction, and excessive autophagy [2, 4, 25–32]. Additionally, phthalate exposures alter the ovarian microenvironment by increasing inflammation and oxidative stress, which contribute to alterations in extracellular matrix (ECM) remodeling that culminate in anovulation and ovarian fibrosis, which are markers of ovarian aging among other age-related pathologies [1, 25, 29, 32, 34, 35, 39, 51]. This article summarizes and discusses the current literature on the effects of phthalate exposures on ovarian aging and the mechanisms thought to underlie phthalate-induced ovarian aging (Table 2).

## Methods

A series of searches were conducted on PubMed. Search terms were selected based on current literature on ovarian and reproductive aging [1–3]. Search terms included (phthalate) AND (reproduction) AND (female) AND (aging), (phthalate) AND (ovary) NOT (review) NOT (co-exposure), (phthalate) AND (ovulation), as well as (phthalate) AND (ovary) AND (x), where x represents the following terms: aging, oxidative stress, mitochondria, mitochondrial damage, immune, inflammation, HPO, hypothalamic-pituitary-gonadal (HPG), apoptosis, autophagy, telomere, fibrosis, senescence, DNA damage, and protein homeostasis. Our initial search returned 179 unique articles. Eligible articles included original research papers published between January 2020 and July 2025 that examined the effects of exposure to any individual phthalate or mixture of phthalates on any measure known to be involved in ovarian aging. Epidemiological studies were excluded, as the epidemiological literature on this topic has previously been reviewed [13, 52, 53]. All animal models were considered for inclusion; however, only articles with findings that may translate to adult human exposure paradigms and focused on mechanisms underlying phthalate-induced toxicity were included. After removing articles that did not fit eligibility criteria, the 36 studies reviewed in this article remained (Table 3).

**Table 3:** Exposure paradigms and main effects of phthalate exposure in reviewed studies

Reference DOI	Model	Phthalate(s)	Exposure (Dose, Route, Duration)	Main Effects
10.1093/biolre/ioae164	CD-1 mice	DEHP	Young adult mice (47 days old) were exposed to DEHP through their chow at 0, 0.15,* 1.5,** or 1500 ppm for 6 months.	- Increased ovarian antioxidant enzyme and *Casp3* expression - Altered ovarian and systemic immune responses - Increased deposition of collagens 1 and 3
10.1093/biolre/ioad137	CD-1 mice	Mix	Young adult mice (47 days old) were exposed to Mix through their chow at 0, 0.15,* 1.5,** or 1500 ppm for 1 or 6 months.	- Increased atresia (1 month) - Decreased ovarian *Casp3* and *Gsr* expression (1 month) - Increased ovarian *Star* expression (1 month) - Inhibited primordial follicle activation (6 months) - Increased serum FSH (6 months) - Decreased serum LH and estradiol (6 months)
10.1016/j.reprotox.2023.108489	CD-1 mice	DEHP or DiNP	Young adult mice (47 days old) were exposed to DEHP or DiNP through their chow at 0, 0.15,* 1.5,** or 1500 ppm for 1 or 6 months.	- Decreased serum FSH (1 month) - Inhibited primordial follicle activation (6 months) - Increased serum FSH (6 months) - Decreased serum LH (6 months)
10.1016/j.tiv.2023.105742	Drosophila melanogaster	DEHP or DiNP	Female flies were exposed to DEHP at 1µM* and 10µM** or DiNP at 1µM** or 10µM through the culture medium. For lifespan assays, flies were chronically exposed. For fertility assays, flies were exposed for 3 days, then moved to control medium to mate and lay eggs.	- Reduced lifespan - Reduced fertility
10.1016/j.envpol.2023.122259	C. elegans	DEHP	Worms were exposed to DEHP at 0, 10,* 100,* 1,000, and 10,000 µg/L through the culture medium for 72 h.	- Reduced lifespan - Reduced fertility - Accelerated onset of infertility - Decreased number of offspring - Decreased relative gonad area
10.1016/j.cbpc.2020.108847	Drosophila melanogaster	DiNP	Female and male flies were exposed to DiNP through their food at either 0, 0.1, 0.2, 0.5, or 1%. After 10 days, parental flies were removed, the number of offspring was recorded, and ovarian morphology was examined.	- Decreased number of offspring - Decreased ovarian volume
10.1038/s41522-025-00742-6	C57BL/6J mice	DEHP	Young adult mice (9 weeks) were orally dosed with vehicle (corn oil) or 300 mg/kg/day DEHP for 16 or 32 days.	- Dysregulated folliculogenesis (16 and 32 days) - Increased serum FSH (16 and 32 days) - Altered serum estradiol and progesterone (16 and 32 days) - Altered gut microbial profile and fecal metabolite profile (16 and 32 days) - Decreased number of offspring (32 days) - Increased atresia (32 days)
10.1016/j.reprotox.2019.12.006	CD-1 mice	DEHP or DiNP	Young adult mice (39–40 days old) were orally dosed with vehicle (corn oil), DEHP at 20 µg/kg/day, * 200 µg/kg/day, ** 20 mg/kg/day, or 200 mg/kg/day, or DiNP at 20 µg/kg/day, ** 100 µg/kg/day, 20 mg/kg/day, or 200 mg/kg/day for 10 days. Following dosing, mice were housed for 12, 15, or 18 months before fertility studies and/or tissue collections.	12 Months post-dosing - Decreased litter size (DEHP) - Increased gestational length (DiNP) - Reduced gestational index and birth rate (DEHP and DiNP) - Altered estrous cyclicity (DEHP and DiNP) - Dysregulated folliculogenesis (DEHP and DiNP) - Increased serum FSH (DEHP and DiNP) - Decreased serum estradiol (DEHP) 15 Months post-dosing - Altered estrous cyclicity (DEHP) - Dysregulated folliculogenesis (DEHP and DiNP) - Increased serum progesterone (DEHP) 18 Months post-dosing - Dysregulated serum estradiol levels (DEHP) - Dysregulated folliculogenesis (DiNP)
10.1093/toxsci/kfad030	CD-1 mice	DEHP, DiNP, or Mix	Young adult mice (47 days old) were exposed to DEHP, DiNP, or Mix at 0, 0.15,* 1.5,** 1500 ppm for 12 months before beginning fertility studies, and mice were cycled at 1, 3, 5, 7, and 11 months of exposure.	- Altered estrous cyclicity (DEHP, DiNP, and Mix) - Decreased gestational index and birth rate (DiNP)
10.1016/j.aquatox.2024.106980	Zebrafish	DBP	Adult zebrafish (4 months old) were exposed to DBP at 50 µg/L in the water for either 0, 2, 4, or 6 weeks.	- Increased oxidative impairment in D-loop region of mitochondrial DNA (6 weeks) - Decreased hatching rate and increased mortality of offspring (4 and 6 weeks) - Decreased ovarian volume (6 weeks)
https://doi.org/10.1002%2Ftox.23121	ICR mice	DEHP	Adolescent mice (4 weeks old) were exposed to vehicle (corn oil) or DEHP at 500 or 1500 mg/kg/day via gavage for 30 days.	- Decreased ovarian organ coefficient - Altered estrous cyclicity - Decreased serum estradiol and progesterone - Dysregulated folliculogenesis - Increased atresia - Induced ovarian oxidative stress - Increased serum IL-1β and TNF-α - Altered gut microbial profile and fecal metabolite profile
10.1016/j.scitotenv.2019.134406	Wistar rats	DEHP	Adult rats (2–3 months old) were orally dosed with vehicle (corn oil) or DEHP at 300, 1000, or 3000 mg/kg/day for 4 weeks.	- Decreased ovarian organ coefficient - Altered estrous cyclicity - Decreased circulating FSH, LH, estradiol, and progesterone - Decreased ovarian expression of steroidogenic enzymes - Induced granulosa cell apoptosis
10.1016/j.tox.2024.153952	Sprague Dawley rats	DEHP	Adolescent rats (4 weeks old) were exposed to vehicle (saline) or DEHP at 0.1*, 1, or 10 mg/kg/day via gavage for 6 weeks.	- Decreased ovarian organ coefficient - Dysregulated folliculogenesis - Increased atresia - Induced ovarian oxidative stress - Increased ovarian collagen 1 deposition
10.1016/j.ecoenv.2023.115534	ICR mice, KGN cells, or primary mouse granulosa cells	DEHP or MEHP	Young adult mice (5 weeks old) were exposed to vehicle (corn oil) or DEHP at 250, 500, or 1000 mg/kg/day via gavage for 30 days. KGN cells were cultured in media containing MEHP at 100, 200, or 400µM for 24 h. Primary mouse granulosa cells were cultured in media containing MEHP at 200µM for 24 h.	- Decreased ovarian organ coefficient - Decreased serum estradiol and progesterone - Dysregulated folliculogenesis - Increased atresia - Induced excessive autophagy (mouse ovary, KGN cells, and primary mouse granulosa cells) - Induced oxidative stress and mitochondrial damage (KGN cells)
10.1016/j.ecoenv.2024.117104	ICR mice or IMGCs	DEHP or MEHP	Prepubertal mice (3 weeks old) were exposed to vehicle (corn oil) or DEHP at 100, 250, or 500 mg/kg/day via gavage for 14 days. IMGCs were cultured in media containing MEHP at 0, 5, 10, 25, 50, 100, 200, 400, 600, or 800µM for 48 h.	- Decreased ovarian organ coefficient - Decreased serum estradiol and progesterone - Induced oxidative stress and ferroptosis (mouse ovary and IMGCs) - Induced mitochondrial dysfunction (IMGCs)
10.1016/j.ecoenv.2023.114625	ICR mice or KGN cells	DEHP	Prepubertal mice (21 days old) were orally dosed with vehicle (corn oil) or DEHP at 2 g/kg/day for 8 days.	- Decreased ovarian organ coefficient - Altered ovarian expression of steroidogenic enzymes - Dysregulated folliculogenesis and decreased serum AMH - Induced granulosa cell pyroptosis - Activated NLRP3 inflammasome in the ovary - Induced ovarian inflammation
10.1016/j.scitotenv.2020.140293	Quail or primary quail granulosa cells	DEHP or MEHP	Prepubertal quail (15 days old) were exposed to vehicle (corn oil) or DEHP at 250, 500, or 1000 mg/kg/day via gavage for 45 days. Primary quail granulosa cells were cultured in media containing MEHP at 50, 100, or 200µM for 24 h.	- Decreased ovarian organ coefficient - Decreased serum FSH and estradiol - Increased serum LH and progesterone - Induced ovarian oxidative stress - Altered ovarian expression of steroidogenic enzymes - Damaged ovarian mitochondria - Activated PPARγ and PPARα (primary quail granulosa cells)
10.1016/j.ecoenv.2024.116679	CD-1 mice or KGN cells	DEHP or MEHP	Young adult mice (5 weeks old) were exposed to DEHP at 0, 5, or 500 mg/kg/day through their food for 4 weeks. KGN cells were cultured in media containing MEHP at 200 or 300µM.	- Altered estrous cyclicity - Dysregulated folliculogenesis and decreased serum AMH - Altered ovarian expression of genes involved in autophagic regulation - Increased follicular apoptosis and atresia - Increased intracellular ROS (mouse ovary and KGN cells) - Induced mitochondrial damage and dysfunction (mouse ovary)
10.1016/j.reprotox.2024.108748	CD-1 mice or primary mouse antral follicles	DEHTP or MEHTP	Adolescent mice (30 days old) were orally dosed with vehicle (corn oil) or DEHTP at 10 µg/kg/day*, 100 µg/kg/day**, or 100 mg/kg/day. Primary mouse antral follicles were cultured in media containing DEHTP or MEHTP at 0.1*, 1*, 10**, or 100 µg/mL for 24–96 h.	Mice - Dysregulated folliculogenesis and increased abundance of abnormal follicles - Altered ovarian expression of steroidogenic enzymes and genes involved in apoptosis Primary mouse antral follicles - Inhibited follicle growth (DEHTP and MEHTP) - Increased Bax/Bcl2 ratio (MEHTP) - Decreased estradiol secretion and increased progesterone secretion (MEHTP) - Altered expression of steroidogenic enzymes (MEHTP)
10.1093/toxsci/kfad064	CD-1 mice	DBP	Young adult mice (35 days old) were orally dosed with vehicle (corn oil) or DBP at 10 µg/kg/day*, 100 µg/kg/day**, or 1000 mg/kg/day for 20–32 days.	- Dysregulated folliculogenesis - Reduced ovarian *Igf1* and *Igf1r* expression - Reduced percentage of oocytes and granulosa cells positive for pIGF1R
https://doi.org/10.1021/acs.jafc.2c08601?urlappend=%3Fref%3DPDF&jav=VoR&rel=cite-as	Quail	DEHP	Prepubertal quail (15 days old) were exposed to vehicle (corn oil) or DEHP at 250, 500, or 750 mg/kg/day via gavage for 45 days.	- Decreased serum estradiol, progesterone, and FSH - Increased serum LH - Induced ovarian mitochondrial dysfunction and autophagy
https://10.2166/wh.2022.140	Rats	DEHP	Adult rats (2 months old) were exposed to vehicle (corn oil) or DEHP at 0.1*, 1.5, or 27.25 mg/kg/day via intraperitoneal injection for 21 days.	- Decreased serum estradiol and progesterone
10.1016/j.envpol.2020.114362	Zebrafish	MEHP	Adult zebrafish (4–6 months old) were exposed to MEHP at 0, 2, 10, or 50 µg/mL through the water for 21 days.	- Increased total estradiol and progesterone
https://10.1016/j.taap.2019.114875	Primary mouse antral follicles	MonoMix	Primary mouse antral follicles were cultured in media containing MonoMix at 0, 0.065,* 0.65,* 6.5,** 65, or 325 µg/mL for 24–96 h.	- Altered estradiol secretion - Increased progesterone secretion - Altered expression of steroidogenic enzymes and PPAR genes - Altered apoptosis - Induced oxidative stress
10.1016/j.reprotox.2024.108737	Primary rat granulosa cells	DEHP	Primary rat granulosa cells were cultured in media containing DEHP at 0 or 400 µM for 24 h.	- Induced oxidative stress and apoptosis - Reduced expression of steroidogenic enzymes - Reduced mitochondrial membrane potential
10.1080/13880209.2023.2249193	KGN cells	DEHP	KGN cells were cultured in media containing DEHP at 0 or 1µM for 24 h.	- Induced oxidative stress and mitochondrial dysfunction
10.1016/j.ecoenv.2022.113898	Kunming mice or primary mouse granulosa cells	DiNP	Adult mice (5 weeks old) were exposed to DiNP at 0, 2, 20, or 200 mg/kg/day via gavage for 14 days. Primary mouse granulosa cells were cultured in media containing DiNP at 0, 100, 200, or 400µM.	- Induced oxidative stress, apoptosis, and autophagy (mouse ovaries and cultured granulosa cells)
10.1016/j.reprotox.2022.04.002	Primary mouse granulosa cells	Phthalate Monoesters (MNP, MiNP, MEHP, MBzP, MBP, MiBP, and MEP)	Primary mouse granulosa cells were cultured in media containing phthalate monoesters at 0, 0.4,* 4,** 40, or 400µM.	- Some phthalate monoesters activate PPARα and/or PPARγ
10.1016/j.reprotox.2025.108938	Primary mouse granulosa cells	MEHHP or EpiMix	Primary mouse granulosa cells were cultured in media containing MEHHP (0, 0.22,* or 22µM or EpiMix at 0, 2,* or 200µM for 24–72 h.	- Cells relied on glycolysis for energy production due to reduction in mitochondrial respiration (MEHHP and phthalate metabolite mixture)
10.1016/j.ecoenv.2023.115686	Kunming mice or KGN cells	DiNP	Adult mice (5 weeks) were exposed to DiNP at 0, 2, 20, or 200 mg/kg/day via gavage for 14 days. KGN cells were cultured in media containing DiNP at 0, 200, 400, or 800µM for 24 h.	- Induced excessive autophagy (mouse ovaries and KGN cells) - Increased DDIT4 levels (mouse ovaries and KGN cells)
10.1016/j.ecoenv.2023.115680	Brachionus plicatilit Müller (1786)	BBP	Rotifers were exposed to BBP through the water at 0, 0.001, 0.01, 0.1, or 1 mg/L for 24–48 h.	- Increased number of autophagosomes in oocytes
10.1016/j.ecoenv.2024.116319	ICR mice or KGN cells	DEHP or MEHP	Young adult mice (4 weeks) were exposed to DEHP at 0, 500, 1000, or 1500 mg/kg/day via gavage for 30 days. KGN cells were cultured in media containing MEHP at 0, 25, 50, 100, 200, 400, or 800µM for 24 h.	- Increased LPS levels in ovaries and sera - Induced systemic inflammation - Induced inflammation, autophagy, and apoptosis (mouse ovaries and KGN cells)
10.1016/j.reprotox.2022.08.004	Primary human granulosa cells	DEHP	Primary human granulosa cells were cultured in media containing DEHP at 0 or 100µM for 24 h.	- Induced apoptosis and mitochondrial fission - Decreased expression of steroidogenic enzymes
10.1016/j.ecoenv.2023.114717	Primary rat granulosa cells	MEHP	Primary rat granulosa cells were cultured in media containing MEHP at 0, 200, 250, 300, or 350µM for 48 h.	- Induced apoptosis - Decreased COX-2 levels
10.1093/toxsci/kfaa170	Primary mouse antral follicles	Mix	Primary mouse antral follicles were cultured in media containing Mix at 0, 1, 10, 100, or 500 µg/mL for 96 h then treated with hCG at 1.5IU/mL. Follicles were observed 18 h post-hCG treatment to assess ovulation. Media and follicles were collected 4, 11, and 18 h post-hCG treatment for further analyses.	- Inhibited ovulation - Increased *Il6* expression - Altered expression of ECM remodelers and steroidogenic enzymes
10.1016/j.envres.2025.121797	3D spheroids derived from ovarian stromal cells of reproductive-aged or post-menopausal women	EpiMix	Spheroids were cultured in media containing EpiMix at 0 or 200µM for 4 days.	Reproductive-aged spheroids - Decreased collagen 6 - Increased collagens 1 and 3 Post-menopausal spheroids - Decreased EMILIN-1 - Decreased fibrillin

An asterisk (*) indicates that the dose is comparable to human adulthood exposure. Two asterisks (**) indicate that the dose is comparable to human occupational exposure. Doses without asterisks are outside of the range of human exposure or from in vitro studies.

### Effects of Phthalates on Endpoints of Reproductive Toxicity and Ovotoxicity

Phthalates have profound effects on the ovary. Mounting evidence suggests that phthalates can target the female reproductive system and accelerate ovarian aging [1, 7, 8]. Some indicators of accelerated ovarian aging include impaired fertility, changes in fertility indices, changes in estrous cyclicity, altered folliculogenesis, changes in HPO signaling, and altered steroidogenesis [15, 54]. The sections below provide a review of the mechanisms underlying phthalate-induced effects on ovarian aging.

#### Effects of Phthalates on Fertility and Estrous Cyclicity

Female fertility gradually decreases with age [54]. Di(2-ethylhexyl) phthalate (DEHP; 10–10,000 µg/L and 1–10µM) and diisononyl phthalate (DiNP; 1–10µM) exposures reduced fertility in C. elegans and female Drosophila, respectively [5, 6]. Further, DEHP (10–10,000 µg/L) reduced the reproductive lifespan of C. elegans [6].

Offspring number can decrease with increased age, and several studies indicate that phthalates reduced offspring number compared to controls [6, 11, 15, 36, 37]. DEHP decreased the number of fetuses in pregnant mice (300 mg/kg/day) and reduced the number of progeny in C. elegans (10–10,000 µg/L) [6, 37]. DiNP exposure (0.5-1% in food) decreased the number of offspring compared to control in Drosophila [36]. Interestingly, mice exposed to DEHP (200 µg/kg/day) exhibited decreased litter size compared to controls 12 months post-exposure [11].

In aging mice, gestational length is increased, embryo implantation is reduced, and embryo resorption is increased relative to younger mice [15]. Phthalate exposure can impact these reproductive outcomes [11, 27, 38]. Exposure to DiNP (100 µg/kg/day and 200 mg/kg/day) increased gestational length compared to controls in mice 12 months post-exposure [11]. DiNP exposure (1.5-1500ppm) for 12 months reduced the birth rate and gestational index (defined as the number of dams that gave birth to live pups divided by the number of pregnant dams) compared to controls in mice [38]. Exposure to DEHP (20 µg/kg/day or 200 mg/kg/day) or DiNP (20 mg/kg/day) also reduced the gestational index and birth rate (20 µg/kg/day DEHP or DiNP) compared to controls in mice 12 months post-exposure [11]. Di-butyl phthalate (DBP; 50 µg/L) decreased the hatching rate and increased the mortality of offspring of exposed female zebrafish compared to controls [27]. Together, these studies demonstrate that phthalate exposure impairs fertility and changes fertility indices in a manner consistent with accelerated reproductive aging.

In rodents, female reproductive aging is characterized by irregular or lengthened estrous cycles, eventually arresting in persistent estrus at the end of the reproductive lifespan [54]. Time spent in estrus typically increases with increased age in rodents [11, 55]. Exposures to DEHP (500-1500 mg/kg/day and 0.15ppm), DiNP (0.15-1.5ppm), or an environmentally relevant phthalate mixture (Mix; 0.15 and 1500ppm; Table 4) increased the time spent in estrus and decreased the time spent in metestrus/diestrus compared to controls in mice, depending on the duration of phthalate exposure [38, 41]. Interestingly, these effects were also seen 12 (20–200 µg/kg/day) and 15 (20 µg/kg/day) months post-DEHP exposure and 12 months post-DiNP exposure (20 µg/kg/day) in mice [11]. One study, however, found that DEHP (1–3 g/kg/day) increased the time spent in metestrus and the total estrous cycle duration compared to controls in rats [45]. Collectively, mounting evidence indicates that phthalate exposure affects estrous cyclicity in rodents in a manner consistent with reproductive aging, and this effect can be seen far beyond the initial exposure window.

**Table 4:** Phthalate and phthalate metabolite mixture compositions

	Chemical Composition	Environmental Relevance
**Mix**	35.2% DEP, 21% DEHP, 14.9% DBP, 8.6% DiBP, 15.1% DiNP, and 5.1% BBP	Developed based on the phthalate metabolite concentrations from the urine of pregnant women from central Illinois [56].
**MonoMix**	36.7% MEP, 19.4% MEHP, 15.3% MBP, 10.2% MiBP, and 10.2% MiNP, and 8.2% MBzP	Based on the phthalate metabolite concentrations from the urine of pregnant women from central Illinois [56].
**EpiMix**	8.1% MEHHP, 65.6% MEP, 1.3% MEHP, 3.1% MEOHP, 6.4% MECPP, 0.8% MCPP, 6.7% MBP, 5.3% MiBP, and 2.6% MBzP	Based on urinary phthalate metabolite levels from women enrolled in the Midlife Women’s Health Study [57–59].

In addition to fertility and cyclicity, phthalates can cause changes in ovarian tissue consistent with accelerated aging. As females age, ovarian volume gradually decreases [60]. Interestingly, DEHP exposure decreased the ovarian organ coefficient in rodents and quails (0.1-3000 mg/kg/day), as well as the relative gonad area compared to controls in C. elegans (10–10,000 µg/L) [6, 32, 33, 42, 45, 47, 61]. Some evidence also indicates that exposure to other phthalates such as DiNP (0.2-1% in food) and DBP (50 µg/L) reduced ovarian volume compared to controls in Drosophila and zebrafish, respectively; however, research in more complex models is necessary to elucidate these connections [27, 36]. It is likely that the decreased ovarian volume observed with phthalate exposure is due to alterations in ovarian folliculogenesis.

#### Effects of Phthalates on Ovarian Follicle Dynamics

DEHP exposure at various doses and exposure paradigms reduced the total number of follicles and the numbers of follicles in different developmental stages compared to controls in rodents. Specifically, DEHP exposure decreased the numbers of primordial (100 µg–2 g/kg/day), primary (100 µg–2 g/kg/day), secondary (0.5–2 g/kg/day), antral (0.5–2 g/kg/day), and total (0.5–1 g/kg/day) follicles compared to control in rodents [32, 33, 37, 41, 42]. Interestingly, DEHP exposure at 5 mg/kg/day increased the number of secondary follicles compared to control [26]. Given that the primordial follicle pool is an indicator of the reproductive lifespan, the DEHP-induced decrease in the number of primordial follicles compared to control indicates that DEHP may induce premature ovarian failure by accelerating the loss of the primordial follicle pool [15]. DEHP exposure also reduced the number of developing follicles compared to control, which is an age-related pathology [15]. Consistent with these observations, DEHP exposure increased the number (1-500 mg/kg/day) and percentage (0.5-500 mg/kg/day) of atretic follicles and reduced serum anti-Müllerian hormone (AMH; 5 mg–2 g/kg/day), a reliable marker of ovarian reserve in mice [26, 33, 37, 41, 42, 49]. Interestingly, DEHP exposure (1500ppm) for 6 months increased the numbers and percentages of primordial follicles and decreased the percentages of preantral and antral follicles, suggesting that chronic high-dose DEHP exposure inhibits primordial follicle activation [4].

Far less research exists on the effects of other phthalates on ovarian folliculogenesis. However, studies show that DiNP exposure for 6 months increased the number and percentage of primordial follicles (1500ppm) and decreased the percentages of preantral (1500ppm) and antral follicles (1.5-1500ppm) compared to control in mice [4]. Mix exposure for 1 month (0.15ppm) increased the numbers of preantral, antral, and atretic follicles, and Mix exposure for 6 months increased the number (1500ppm) and percentage (1.5-1500ppm) of primordial follicles and decreased the percentages of preantral (1500ppm) and antral (1.5-1500ppm) follicles compared to controls in mice [2]. Collectively, these studies indicate that chronic DiNP and Mix exposures may inhibit primordial follicle activation in mice.

Studies also show that exposure to di(2-ethylhexyl) terephthalate (DEHTP) decreased the number and percentage of primordial follicles (100 mg/kg/day), increased the number (100 mg/kg/day) and percentage (100 µg-100 mg/kg/day) of primary follicles, decreased the percentage of preantral follicles (100 µg/kg/day), and increased the number and percentage of abnormal follicles (100 mg/kg/day) compared to control in mice [43]. Similar to the effects of acute DEHP exposure on the follicle pool, DEHTP exposure reduced primordial follicles, which may lead to premature ovarian failure. DEHP and DiNP (20 µg-200 mg/kg/day) exposures can also affect follicle dynamics and the ovarian follicle pool 12–18 months post-exposure [11]. Exposure to DBP also decreased primordial (100 µg/kg/day and 1 g/kg/day), primary (100 µg/kg/day), and total follicle (100 µg/kg/day) numbers compared to control in mice [40].

Although the mechanisms by which phthalates alter folliculogenesis are unclear, one study suggests that reductions in the insulin-like growth factor 1 (IGF-1) pathway are responsible for DBP-induced changes in folliculogenesis [40]. Specifically, DBP exposure (100 µg/kg/day) reduced ovarian *Igf1* and *Igf1r* expression and decreased the percentage of phosphorylated IGF-1 receptor (pIGF-1R) positive oocytes and granulosa cells compared to control [40]. Inhibition of IGF-1R signaling can induce phenotypes associated with accelerated ovarian aging, such as inhibiting the progression of antral follicles to the preovulatory stage, reducing serum estrogen, impairing ovarian FSH action, and decreasing overall fertility [15, 40]. It is possible that changes in the IGF-1 pathway are, in part, responsible for other phthalate-induced changes in follicle development and age-associated phenotypes, but this possibility has not been tested.

#### Effects of Phthalates on Gonadotropin Hormone Levels and Ovarian Steroidogenesis

One of the primary functions of the developing follicle is the synthesis of sex steroid hormones. Both folliculogenesis and steroidogenesis are tightly regulated by the HPO axis. The pituitary communicates with the ovary through the gonadotropin hormones, FSH and LH, and the ovary communicates with the hypothalamus and pituitary through sex steroid hormones [2]. This communication becomes dysregulated with increased age, leading to excessive FSH secretion and dysregulated LH secretion [15]. Phthalates have differing effects on serum concentrations of FSH and LH. DEHP exposure increased serum FSH in mice (300 mg/kg/day), decreased serum FSH (1 g/kg/day) and LH (1–3 g/kg/day) in rats, and decreased serum FSH (500-1000 mg/kg/day) and increased serum LH (250-1000 mg/kg/day) in quails compared to controls [37, 45, 46, 61]. DEHP (0.15ppm) or DiNP (1.5ppm) exposure for 1 month decreased serum FSH, DEHP (1500ppm), DiNP (1.5ppm), or Mix (0.15ppm) exposure for 6 months increased circulating FSH, and DEHP (0.15-1.5ppm), DiNP (1.5-1500ppm), and Mix (0.15-1500ppm) exposure for 6 months decreased circulating LH compared to controls in mice [2, 4]. Similar to the effects of phthalates on folliculogenesis, the effects of phthalates on serum FSH and LH concentrations can last far beyond the initial window of exposure. Exposure to DEHP (20 µg/kg/day) or DiNP (20 mg/kg/day) increased serum FSH levels compared to controls 12 months post-dosing [11]. These changes in serum gonadotropin levels may contribute to phthalate-induced changes in folliculogenesis and steroidogenesis and are likely indicative of age-associated HPO dysregulation.

HPO dysregulation contributes to decreased circulating estradiol and progesterone levels in aged individuals [15]. Phthalates have profound effects on ovarian steroidogenesis that result in significant changes to circulating estradiol and progesterone levels. DEHP exposure decreased circulating estradiol (1.5–3 g/kg/day) and progesterone (1.5-500 mg/kg/day) in rodents, with the exception that exposure at 300 mg/kg/day for 16 days increased circulating estradiol and progesterone compared to control in mice [32, 37, 41, 45, 47, 48]. These effects can last far beyond the initial exposure window. Mice exposed to DEHP exhibited decreased serum estradiol at 12 (20 µg/kg/day) and 18 (100 µg/kg/day) months post-dosing, increased progesterone (200 µg/kg/day) at 15 months post-dosing, and increased estradiol (200 mg/kg/day) at 18 months post-dosing compared to controls [11]. DEHP exposure also reduced serum estradiol (250-1000 mg/kg/day) and progesterone (250-1000 mg/kg/day) compared to control in quails [46, 61]. Exposure to Mix (1500ppm) decreased serum estradiol compared to control in mice [2]. Interestingly, exposure to phthalate metabolites can produce opposite effects compared to their parent compounds. Mono(2-ethylhexyl) phthalate (MEHP) exposure in zebrafish increased total estradiol (2–50 µg/mL) and progesterone (2–50 µg/mL) compared to control [44]. An environmentally relevant phthalate metabolite mixture (MonoMix; Table 4) increased estradiol (6.5–65 µg/mL) and progesterone (65–325 µg/mL) secretion after 24 h and decreased estradiol (325 µg/mL) secretion and increased progesterone (6.5–325 µg/mL) secretion compared to controls after 96 h in cultured mouse antral follicles [50]. The effects of phthalates on circulating sex steroid hormones may be explained by phthalate-induced HPO dysregulation and alterations in steroidogenic enzyme expression observed in numerous studies [2, 39, 42, 43, 50, 61].

### Effects of Phthalates on Oxidative Stress Pathways in the Ovary

Oxidative stress increases during ovarian aging, and phthalates are known to cause oxidative stress, which is characterized by the imbalance between reactive oxygen species (ROS) production and accumulation and the ability of the cell to detoxify ROS [1, 62]. DEHP increased total ROS in rat ovaries (100 µg and 10 mg/kg/day), rat granulosa cells (400µM), mouse oocytes (5 or 500 mg/kg/day), and a human granulosa cell-like tumor cell line (KGN; 1µM) compared to controls [25, 26, 33, 39]. MEHP increased intracellular ROS in KGN cells (100–400µM) and in an immortalized mouse granulosa cell line (IMGC; 200µM) compared to controls [26, 32, 47].

Cells have an antioxidant defense system that protects them from oxidative damage. This antioxidant defense system is composed of antioxidant enzymes such as superoxide dismutase (SOD) and catalase (CAT) [62]. Phthalates can alter the expression and activity of these enzymes. DEHP increased *Cat* and *Sod1* expression (1500ppm) as well as SOD protein levels (500-1500 mg/kg/day) in mouse ovaries compared to controls; however, MEHP (100–200µM) decreased SOD in KGN cells, and DEHP decreased SOD in quail ovaries (500-1000 mg/kg/day) compared to controls [4, 32, 41, 61]. MonoMix (65 µg/L) also increased *Sod1* expression in mouse antral follicles compared to controls [50]. Interestingly, DiNP decreased SOD activity in mouse ovaries (20-200 mg/kg/day) and granulosa cells (200–400µM), which may contribute to ROS accumulation [29]. MonoMix altered *Cat* expression in mouse antral follicles compared to controls [50]. Phthalates also increased malondialdehyde (MDA), a byproduct of lipid peroxidation compared to controls [62]. DEHP increased MDA in ovaries of rats (1-10 mg/kg/day), mice (100-1500 mg/kg/day), quails (500-1000 mg/kg/day), and KGN cells (1µM) compared to controls [25, 33, 41, 47, 61]. MEHP increased MDA content in cultured KGN cells (100–200µM) and IMGCs (200µM) compared to controls [32, 47]. Finally, DiNP increased MDA content in mouse ovaries (200 mg/kg/day) and granulosa cells (200–400µM) compared to controls [29]. Taken together, overwhelming evidence indicates that phthalates cause oxidative stress in the ovary, which contributes to phthalate-induced accelerated ovarian aging in a variety of ways.

Another important antioxidant defense is the glutathione system. The antioxidant enzyme, glutathione peroxidase (GPX) controls ROS by converting reduced glutathione (GSH) to oxidized glutathione (GSSG) [2, 62, 63]. The enzyme, glutathione reductase (GSR) catalyzes the reduction of GSSG back to GSH [2, 63]. High GSH, GPX, and GSR levels are indicative of high antioxidant capacity [63]. Phthalates can alter this system significantly. DEHP (250-1000 mg/kg/day) increased GSH concentration and decreased GPX activity in quail ovaries compared to controls, and MEHP (100–200µM) decreased GSH in KGN cells compared to controls [32, 61]. DEHP (250 mg/kg/day) also reduced GPX4 in mouse ovaries, and MEHP (200µM) decreased GPX4 in IMGCs compared to controls [47]. DiNP decreased GPX activity in mouse ovaries (2-200 mg/kg/day) and granulosa cells (400µM) and decreased GSH content in mouse ovaries (20-200 mg/kg/day) and granulosa cells (100–400µM) compared to controls [29]. Mix (0.15ppm) decreased *Gsr* expression in mouse ovaries compared to controls, and MonoMix decreased *Gpx* (325 µg/L) and *Gsr* (0.065–325 µg/L) expression in mouse antral follicles compared to controls [2, 50]. Interestingly, GSH is important for inhibiting ferroptosis, a mechanism of cell death that contributes to follicular atresia [2]. One study suggests that DEHP (100-500 mg/kg/day) induced ferroptosis in the mouse ovary through an Nrf2-mediated signaling pathway [47]. Collectively, these data indicate that phthalates dysregulate the glutathione antioxidant system, which contributes to oxidative stress and phthalate-mediated ferroptosis.

Phthalates are known to activate peroxisome proliferator activated receptors (PPAR), which are primarily responsible for inducing oxidative stress among other functions [33, 64]. A recent study found that some phthalate monoesters act through PPARα and/or PPARγ in mouse granulosa cells [65]. PPARγ appears to be a target of DEHP and its metabolite MEHP. DEHP exposure increased PPARγ (100 µg-10 mg/kg/day) as well as retinoid X receptor α (RXRα) and fatty acid translocase (CD36; 1-10 mg/kg/day) in rat ovaries compared to controls [33]. MEHP exposure decreased cytoplasmic (100–200µM) and increased nuclear (50–200µM) PPARγ, increased *Pparγ* (50–200µM) and fatty acid-binding protein (*Fabp*; 100–200µM) expression, and altered *Rxrγ* (50–200µM) expression in quail granulosa cells compared to controls [61]. Phthalates may also target PPARα. MEHP decreased cytoplasmic and increased nuclear PPARα (100–200µM), increased *Rxrα* (100–200µM), and altered *Pparα* (50 and 200µM) expression in quail granulosa cells compared to controls [61]. MonoMix exposure can also alter expression of PPAR genes. MonoMix decreased *Pparα* (325 µg/L), increased *Ppard* (65–325 µg/L), and altered *Pparγ* (0.065–325 µg/L) expression in mouse antral follicles compared to controls [50]. Collectively, these studies suggest that phthalate-induced ovarian oxidative stress may be mediated by PPAR activation.

### Phthalates Induce Mitochondrial Dysfunction, Autophagy, and Apoptosis in the Ovary

#### Effects of Phthalates on Mitochondrial Dysfunction in the Ovary

Phthalate-induced oxidative stress may mediate age-related changes in the ovary, such as mitochondrial dysfunction, excessive autophagy, and increased apoptosis. Several studies show that DEHP or MEHP exposure can cause mitochondrial dysfunction, which contributes to the aging phenotype [66]. MEHP (200µM) increased the number of abnormal mitochondria in IMGCs compared to controls [47]. DEHP increased mitochondrial swelling in quail granulosa cells (500-750 mg/kg/day) and mouse ovaries (5 mg/kg/day) compared to controls [26, 46]. Interestingly, mitochondria of senescent ovarian cells exhibited increased mitochondrial mass, suggesting that the DEHP-induced mitochondrial swelling may be age-related [66]. DEHP (1µM) also decreased the mitochondrial DNA copy number in KGN cells compared to controls, another phenotype consistent with ovarian aging [25, 66]. DEHP reduced the abundance of mitochondrial cristae in the ovaries of quails (250-1000 mg/kg/day) and mice (5 mg/kg/day) compared to controls, and MEHP (200µM) reduced the number of mitochondrial cristae in IMGCs compared to controls [26, 47, 61]. Consistent with these observations, DEHP reduced the mitochondrial membrane potential in KGN cells (1µM), rat granulosa cells (400µM), and mouse oocytes (5 or 500 mg/kg/day) compared to controls, and MEHP (200µM) reduced the mitochondrial membrane potential in IMGCs compared to controls [25, 26, 39, 47]. Interestingly, one study found that mouse granulosa cells treated with mono-(2-ethyl-5-hydroxyhexyl phthalate (MEHHP; 0.22-22µM) or an epidemiologically relevant mixture of phthalate metabolites (EpiMix; 2µM; Table 4) rely on glycolysis for energy production due to reduction in mitochondrial respiration [67].

During aging, mitochondrial quality declines, which involves dysregulation of mitochondrial biogenesis, fusion, and fission [66]. DEHP increased levels of some proteins involved in mitochondrial fusion and fission, optic atrophy protein 1 (OPA1), mitofusin (MFN) 1 and 2, and retinitis pigmentosa 1 (RP1) in quail ovaries (250-750 mg/kg/day) and decreased expression of peroxisome proliferator-activate receptor coactivator 1α (*Pgc1α*), a gene involved in mitochondrial biogenesis, in mouse ovaries (5 mg/kg/day) compared to controls [26, 46]. Taken together, these data indicate that phthalates cause mitochondrial dysfunction, which may be mediated by oxidative stress and contribute to phthalate-induced apoptosis.

#### Effects of Phthalates on Autophagy in the Ovary

Autophagy is a process of intracellular organelle and macromolecule degradation that generally protects the cell from pathogenic agents [68]. Excessive ROS can induce autophagy in ovarian cells as the cells attempt to clear ROS and reduce oxidative stress [69]. Excessive autophagy can result in cell death, which may be mediated through protein kinase B (Akt)-mammalian target of rapamycin (mTOR) pathway inhibition [69]. DNA damage-inducible transcript 4 (DDIT4) promotes autophagy by inhibiting mTOR signaling [70]. Interestingly, DiNP exposure increased DDIT4 levels in KGN cells (400–800µM) and mouse ovaries (2-200 mg/kg/day) and increased *Ddit4* expression in KGN cells (800µM) compared to controls [31]. DEHP (5 and 500 mg/kg/day), MEHP (200–300µM), and DiNP (2-200 mg/kg/day and 800µM) altered protein and mRNA levels of autophagic regulators, such as Parkin, C/EBP homologous protein (CHOP), glucose-regulated protein 78 (GRP78), X-box binding protein 1 (XBP1), activating transcription factor (ATF) 4 and 6, and protein kinase RNA-like endoplasmic reticulum kinase (PERK) compared to controls, and autophagic dysregulation is implicated in aging [26, 31, 68, 71].

Execution of autophagy involves formation of an autophagosome, followed by fusion with a lysosome to form an autolysosome that then degrades cytoplasmic components using lysosomal hydrolases [68]. DiNP increased the presence of autophagic vacuoles in mouse granulosa cells (400µM) and KGN cells (800µM), benzyl butyl phthalate (BBP; 1 mg/L) increased the number of autophagosomes in rotifer oocytes, and MEHP (100–200µM) increased the number of autophagosomes and autolysosomes in KGN cells compared to controls [29, 31, 51]. These findings suggest that phthalates increase autophagy in the ovary. Interestingly, autophagy plays a role in inhibiting primordial follicle activation, a phenotype observed with some phthalate exposures [2, 4, 69].

Beclin-1 and autophagy related genes (ATGs) are key elements of autophagic machinery, as they guide autophagosome formation and cellular cargo breakdown [69]. DEHP increased Beclin-1 (250-1000 mg/kg/day) and ATG5 (500-1000 mg/kg/day) levels in mice, and MEHP increased Beclin1 (200–400µM), ATG5 (100–400µM), and total ATG (400–800µM) levels in KGN cells compared to controls [30, 32]. DiNP increased Beclin-1 in mouse ovaries (2-200 mg/kg/day) and granulosa cells (200–400µM), and it increased ATG5 in mouse ovaries (2-200 mg/kg/day), mouse granulosa cells (100–400µM), and KGN cells (800µM) compared to controls [29, 31].

Microtubule-associated protein light chain 3 (LC3)-II assists in the initiation and nucleation of the phagophore to form an autophagosome, and an increased LC3-II/LC3-I ratio indicates increased autophagy [69, 72]. DEHP increased the LC3-II/LC3-I ratio in mouse ovaries (500-1000 mg/kg/day) and increased LC3B levels in quail ovaries (500-750 mg/kg/day), and MEHP increased the LC3-II/LC3-I ratio in KGN cells (200–800µM) compared to controls [30, 32, 46]. DiNP increased the LC3-II/LC3-I ratio in mouse ovaries (2-200 mg/kg/day) and granulosa cells (100–400µM), increased LC3B in mouse ovaries (2-200 mg/kg/day), and increased LC3-II in KGN cells (800µM) compared to controls [29, 31]. Together, these data indicate that phthalate exposure induces excessive autophagy in the ovary, which may increase autophagic cell death.

Sequestosome-1 (P62) is a ubiquitin binding protein that binds cellular waste designated for autophagic degradation [73]. P62 also binds LC3 to link the cellular waste to the autophagosome, which ultimately allows degradation of the waste and the P62 protein itself through autophagy [73]. DEHP increased P62 levels in mouse (250-1000 mg/kg/day) and quail (500-750 mg/kg/day) ovaries, and MEHP increased P62 levels in KGN cells (100–400µM) compared to controls [30, 32, 46]. Interestingly, DiNP decreased P62 levels in KGN cells (800µM) compared to control [31]. Elevated P62 levels indicate that the cell may have more cellular waste than the cell is capable of breaking down through autophagy, which may contribute to autophagic cell death.

#### Effects of Phthalates on Apoptosis in the Ovary

Phthalates significantly increase apoptosis in the ovary [26, 28, 45, 74]. DEHP increased follicular apoptosis in mice (5 or 500 mg/kg/day), increased granulosa cell apoptosis in rats (1–3 g), and increased the percentage of apoptotic cells in primary human granulosa cells (100µM) compared to controls [26, 28, 45]. MEHP increased the apoptosis rate (200–350µM) and decreased cyclooxygenase-2 (COX2) expression at the transcriptional and translational levels (250–350µM) in rat granulosa cells compared to controls [74]. Interestingly, COX2 inhibition can increase apoptosis, and the aforementioned study demonstrated that COX2 overexpression alleviated MEHP-induced apoptosis [74].

The intrinsic pathway of apoptosis is regulated by the B-cell lymphoma-2 (BCL-2) protein family [75]. The effector molecule, Bcl-2-associated protein (BAX), forms pores in the mitochondrial membrane when uninhibited, allowing the release of cytochrome C and subsequent caspase driven apoptosis [75]. Phthalates can increase the abundance and alter the expression of BAX [28, 29, 39, 43, 50, 74]. Specifically, DEHP increased BAX in cultured rat (400µM) and primary human (100µM) granulosa cells compared to controls [28, 39]. MEHP increased *Bax* (350µM) in rat granulosa cells compared to controls [74]. DiNP increased BAX in mouse ovaries (2-200 mg/kg/day) and granulosa cells (100–400µM) compared to controls [29]. MonoMix increased *Bax* at 325µM and decreased *Bax* at 65µM in mouse antral follicles compared to controls [50]. DEHTP (100 mg/kg/day) exposure decreased *Bax* in mouse ovaries compared to control [43].

The anti-apoptotic molecule, BCL-2, sequesters BAX, inhibiting mitochondrial pore formation and thus, inhibiting apoptosis [75]. Phthalate exposure decreased abundance and altered expression of BCL2 [28, 29, 32, 39, 43, 50, 74]. Specifically, DEHP decreased BCL-2 levels in primary human granulosa cells (100µM) and mouse ovaries (500-1000 mg/kg/day) and *Bcl-2* expression in rat granulosa cells (400µM), and MEHP decreased BCL2 abundance (250–300µM) and expression (250–350µM) in rat granulosa cells compared to controls [28, 32, 39, 74]. DiNP decreased BCL-2 in mouse ovaries (2-200 mg/kg/day) and granulosa cells (100–400µM) compared to controls [29]. DEHTP (100 mg/kg/day) decreased *Bcl-2* as well as BCL-2-associated death promoter (*Bad*), an inhibitor of anti-apoptotic molecules, in mouse ovaries compared to controls [43]. MonoMix decreased *Bcl-2* (325 µg/mL) and *Bcl2l10* (0.065–325 µg/mL) and increased *Bcl-2* (0.065 µg/mL) in mouse antral follicles compared to controls [50].

Due to the interactions between BAX and BCL-2, the BAX/BCL-2 ratio offers significant insight into the apoptotic state of the tissue, with a higher BAX/BCL-2 ratio indicating increased apoptosis [75]. DEHP increased the BAX/BCL-2 ratio in mouse (500-1500 mg/kg/day) and rat (300-3000 mg/kg/day) ovaries and primary human granulosa cells (100µM), and MEHP increased the BAX/BCL-2 ratio in rat granulosa cells (300µM) and KGN cells (100–800µM) compared to controls [28, 30, 45, 74]. Mono(2-ethylhexyl) terephthalate (MEHTP) increased the *Bax/Bcl-2* expression ratio (10–100 µg/mL) compared to control in mouse antral follicles [43]. Consistent with these observations, DEHP (100µM) increased cytochrome c in human primary granulosa cells [28]. Together, these data indicate that phthalate exposure increases apoptosis in the ovary, which may contribute to accelerated ovarian aging through increased atresia, leading to early depletion of the follicle pool.

Apoptosis is characterized by caspase-dependent proteolysis [75]. In the intrinsic apoptosis pathway, cytochrome c binds to apoptotic protease activating factor 1 (APAF-1) to form the apoptosome, which activates caspase 9, subsequently activating the effector caspases, caspase 3 and caspase 7, which disassemble the cell [75]. In general, phthalate exposure increased levels of caspase 3 and caspase 7 in various model systems [4, 28, 30, 39, 74]. Specifically, DEHP increased *Casp3* in mice (1500ppm) and rat granulosa cells (400µM) and increased the caspase 3/pro-caspase 3 ratio in primary human granulosa cells (100µM) and mouse ovaries (500-1500 mg/kg/day), and MEHP increased caspase 3 (200–350µM) and caspase 7 (250–350µM) in rat granulosa cells and increased the caspase 3/pro-caspase 3 ratio in KGN cells (200–800µM) compared to controls [2, 4, 28–30, 39, 43, 50, 74]. DiNP increased caspase 3 levels in mouse ovaries (2-200 mg/kg/day) and granulosa cells (200–400µM) compared to controls [29]. Interestingly, Mix decreased *Casp3* expression in mouse ovaries (0.15 and 1500ppm), and MonoMix (65–325µM) and DEHTP (1 µg/L) decreased *Casp3* expression in mouse antral follicles compared to controls [2, 43, 50]. These data indicate that phthalates induce apoptosis through the intrinsic pathway.

Growing evidence suggests researchers should also consider the extrinsic apoptosis pathway when investigating the effects of phthalates on apoptosis in the ovary. Caspase 8 activation is indicative of apoptosis occurring through the extrinsic pathway [75]. Caspase 8 cleaves BH3 interacting domain death agonist (BID), which translocates to the mitochondria, stimulating the intrinsic apoptosis pathway [75]. DiNP increased caspase 8 levels in mouse granulosa cells (200–400µM) and ovaries (2-200 mg/kg/day), MEHTP increased *Casp8* expression (1 µg/mL) in mouse antral follicles, and MonoMix decreased *Casp8* in mouse antral follicles (0.65–325 µg/mL) compared to controls [29, 43, 50]. Interestingly, MonoMix had differing effects on *Bid* expression in mouse antral follicles depending on dose, increasing *Bid* expression at 325 µg/mL and decreasing *Bid* at 0.65 µg/mL, and DEHTP (100 mg/kg/day) decreased *Bid* expression in mouse ovaries compared to controls [43, 50]. These results suggest that phthalate-induced apoptosis may occur through the extrinsic pathway, and future research should investigate the effects of phthalates on the extrinsic apoptosis pathway.

Crosstalk between apoptosis, ferroptosis, and autophagy plays a role in initiation of atresia [2]. Phthalate-induced oxidative stress may mediate the phthalate-induced excessive autophagy observed in numerous studies and induce ferroptosis in the ovary. Further, phthalate-induced mitochondrial dysfunction mediated by oxidative stress may contribute to phthalate-induced apoptosis. Together, the increases in autophagy, ferroptosis, and apoptosis may contribute to excessive follicular atresia that accelerates depletion of the follicle pool and thus accelerates ovarian aging.

### The Effects of Phthalates on Ovarian Inflammation, Ovulation, and Ovarian Fibrosis

One possible mechanism by which phthalates accelerate ovarian aging involves the activation of the NLR family pyrin domain containing 3 (NLRP3) inflammasome through oxidative stress, subsequently promoting local and systemic inflammation, which can ultimately contribute to defects in ECM remodeling, leading to age-related pathologies such as fibrosis and anovulation. It is well established that phthalates cause oxidative stress and that oxidative stress can both directly and indirectly activate the NLRP3 inflammasome to promote inflammation [25, 26, 32, 33, 39, 47, 76].

#### Effects of Phthalates on Ovarian Inflammation

Tumor necrosis factor α (TNFα) is a cytokine that plays a crucial role in inducing inflammation and has an inhibitory role in ovarian processes such as folliculogenesis and steroidogenesis. Specifically, excessive ROS in oocytes and granulosa cells induced the release of TNFα [77]. TNFα inhibited the FSH-stimulated secretion of estradiol from granulosa cells, likely reducing FSH and LH driven granulosa cell differentiation [78]. Interestingly, TNFα levels are associated with reproductive age [78, 79]. Levels of TNFα are significantly higher in the ovaries of aged mice compared with young mice, and a greater concentration of TNFα is present in the follicular fluid of women with diminished ovarian reserve compared with controls [78, 79]. DEHP (500-2000 mg/kg/day) increased TNFα levels in mouse ovaries and plasma, and MEHP (200µM) increased *Tnfα* expression in KGN cells compared to controls [30, 41, 42]. Interestingly, TNFα is also a transcriptional regulator of components of the NLRP3 inflammasome, acting through the nuclear factor kappa-light-chain-enhancer of activated B cells (NF-κB) pathway [80]. More research is necessary to elucidate the connection between phthalate exposure and elevated TNFα levels; however, the molecule is a promising potential target for treatment of phthalate-induced ovarian dysfunction and accelerated ovarian aging.

The NLRP3 inflammasome is an important activator of immune activity in the ovary [81]. The inflammasome and its associated molecules, interleukin (IL)1β, IL18, and caspase 1 (Casp1), are implicated in numerous adverse reproductive outcomes in females, including age-associated ovarian inflammation and loss of fertility, depletion of the follicular reserve, POI, ovarian tumors, and polycystic ovarian syndrome [82–87]. DEHP (2 g/kg/day) exposure elevated the levels of NLRP3, IL1β/pro-IL1β, Casp1/pro-Casp1 in mouse ovaries and KGN cells and increased serum IL1β levels compared to controls [42]. MEHP (200µM) increased IL1β at both the transcriptional and translational levels compared to control in KGN cells [30]. DEHP (500-1500 mg/kg/day) increased ovarian and serum IL1β levels in mice compared to controls [30, 41]. Interestingly, chronic DEHP exposure at 1500 ppm decreased ovarian *Il1b* expression compared to control in mice [1]. Overall, these studies suggest that high-dose DEHP exposure might induce activation of the NLRP3 inflammasome, whereas activation of the NLRP3 inflammasome may subside with chronic DEHP exposure.

Exposures to phthalates have many effects on ovarian and systemic inflammation, as evidenced by the phthalate-induced increases in pro-inflammatory interleukins, decreases in anti-inflammatory interleukins, and dysregulation of ovarian and systemic cytokine levels [1, 30, 35]. Exposure to DEHP (500-1500 mg/kg/day) for 30 days increased the levels of the pro-inflammatory cytokine, IL6, in the whole ovary, granulosa cells, and sera of mice and decreased the levels of the anti-inflammatory cytokine, IL10, in granulosa cells of exposed mice compared to controls [30]. MEHP (200µM) exposure increased expression of the pro-inflammatory *Il6* and *Il8*, and decreased expression of *Il10* in KGN cells compared to controls [30]. MEHP (200µM) also increased IL6 levels in KGN cells compared to controls [30]. Mix (100–500 µg/mL) exposure decreased *Il6* expression in mouse antral follicles compared to controls [35]. Finally, a study examining the effects of chronic DEHP exposure (1.5 and 1500ppm) revealed dysregulated ovarian and systemic cytokine profiles in DEHP exposed mice compared to controls [1]. Some molecules that were aberrantly expressed in both the ovaries and sera in response to DEHP included C-X-C motif chemokine ligand 16 (CXCL16), retinol binding protein 4 (RBP4), vascular cell adhesion molecule-1 (VCAM-1), adiponectin, and C-reactive protein (CRP) [1]. Interestingly, DEHP exposure downregulated RBP4, VCAM-1, adiponectin, and CRP in the ovary and upregulated the same molecules in the sera, suggesting that the ovary has a DEHP-induced immune response independent of the systemic immune response [1]. Overall, it is evident that phthalate exposure can increase ovarian and systemic inflammation and cause an ovary-specific immune response.

#### Effects of Phthalates on Ovarian Extracellular Matrix Remodeling

A growing body of evidence indicates that phthalate exposure can induce ECM remodeling, resulting in fibrosis and anovulation in mammalian ovaries [1, 33–35]. Interestingly, chronic inflammation and ECM remodeling play a role in both fibrosis and anovulation. Cytokines play a regulatory role in matrix metalloprotease (MMP) expression in the ovary throughout the estrous cycle, and they are especially important during ovulation [88]. The ovulatory LH surge increases expression of ovarian cytokines such as interleukins and tumor necrosis factor, which can either act independently or as a part of a signaling pathway to regulate MMPs and the tissue inhibitors of metalloprotease (TIMPs) [88]. Perhaps, the phthalate-induced dysregulation of ovarian and systemic cytokine levels contributes to dysregulation of MMP and TIMP levels, leading to fibrosis and anovulation, and ultimately accelerating ovarian aging.

One study investigating the effects of Mix on ovulation in mouse antral follicles found that Mix upregulated *Mmp14* (10–500 µg/mL), *Mmp16* (1–100 µg/mL), and *Mmp19* (500 µg/mL) and downregulated *Timp1* (500 µg/mL) expression following an ovulatory stimulus (hCG treatment) [35]. Mix upregulated *Mmp9* 11 h post-hCG treatment (1–500 µg/mL) despite downregulating the same gene 4 h post-treatment (100–500 µg/mL) [35]. Upregulation of *Timp1* post-ovulation is crucial to terminate MMP activity and promote follicle repair [35]. Given that Mix (1–500 µg/mL) exposure reduced ovulation rates in cultured antral follicles compared to controls, it is possible that the Mix-induced changes in the expression of genes that modulate ECM deposition are due to the lack of *Timp1* upregulation stemming from anovulation [35]. It is also possible that the Mix-induced change in *Timp1* expression is due to an alternate mechanism and that changes in *Timp1* expression may lead to the elevated MMP expression, which is associated with increases in proapoptotic factors that may contribute to peri-ovulatory atresia, ultimately resulting in reduced ovulation in Mix-exposed follicles compared to controls. Interestingly, a study investigating the effects of chronic DEHP exposure on reproductive function found that MMP2 and MMP3 were downregulated in the sera of mice exposed to 1500 ppm DEHP through the food for 6 months compared to controls [1]. Given that anovulation and alterations in tissue remodeling pathways are important hallmarks of ovarian aging, these studies show that Mix can cause anovulation in cultured antral follicles and disrupt expression of tissue remodeling molecules, suggesting accelerated ovarian aging [89]. More work should be done to further elucidate the effects of phthalates on ovulation-related tissue remodeling and accelerated ovarian aging.

Ovarian fibrosis is characterized by an excess deposition of ECM components such as collagen in the ovarian stroma [1]. Chronic DEHP exposure for 6 months increased the deposition of collagens 1 (0.15-1.5ppm) and 3 (1.5ppm) in the mouse ovary compared to controls [1]. DEHP (100 µg-10 mg/kg/day) for 6 weeks increased deposition of collagen 1 in the ovary compared to controls in adolescent rat ovaries [33]. In addition, a study investigating the effects of EpiMix on ovarian ECM composition using a 3-dimensional ovarian spheroid model created using human ovarian stromal tissue from either reproductive aged or post-menopausal women found that exposure to EpiMix (200µM) in culture for 4 days reduced collagen 6 and increased collagens 1 and 3 in reproductive aged spheroids [34]. Collagen 6 is capable of inhibiting apoptosis and reducing oxidative stress [90]. The EpiMix-induced reduction in collagen 6 suggests that the spheroid loses some of its regulatory control of these processes, which may exacerbate the negative effects of phthalates on the system. The EpiMix-induced changes in collagen deposition without concurrent changes in elastin microfibril interfacer 1 (EMILIN-1) and fibrillin-1 suggest that EpiMix is driving the ECM toward a profibrotic state, which may ultimately impact reproductive function [34]. Interestingly, EpiMix (200µM) exposure reduced EMILIN-1 and fibrillin-1 in post-menopausal spheroids, indicating reduced tissue elasticity compared to controls [34]. It is possible that EpiMix-induced ECM remodeling is caused by phthalate-induced alterations in MMP activity [34]. Previous studies on phthalate exposures support this hypothesis [1, 35].

### Phthalate-Induced Intestinal Microbiome Dysregulation is Linked to Phthalate-Induced Ovarian Aging

The intestinal microbiome has an interesting link to reproductive dysfunction and aging that may elucidate some of the phthalate-induced effects on ovarian dysfunction and aging. Several studies suggest that the effects of phthalates on the ovary are mediated in part by phthalate-induced gut microbial dysbiosis, resulting in alterations in estrogen signaling and compromising gut barrier integrity, leading to increased LPS levels in circulation that can then target the ovary [30, 37, 41].

The intestinal microbiome is intimately linked to endocrine signaling in female mammals [16]. Although estrogen signaling can modulate the microbiome, the microbiome is also an important regulator of circulating estrogen [16]. As a result of this interplay, the female reproductive system is influenced by changes in the gut microbiome, and some of these changes can even lead to accelerated ovarian aging [16]. Estrogen mimicking compounds, such as phthalates, can modulate the mouse microbiome, which in turn can interfere with proper ovarian function [41]. Specifically, studies show that DEHP can significantly alter the gut microbiota and fecal metabolite profile in a manner that may contribute to accelerated ovarian aging [37, 41]. DBP has also been shown to induce gut bacterial dysbiosis [91].

β-glucuronidase (GUS) is an enzyme produced by some bacterial species that can reactivate inactive estrogen previously designated for transport to the intestine and excretion [16]. Alterations in GUS activity, driven by gut microbial dysbiosis, can contribute to the development of estrogen-related disorders by activating or participating in the circulation of estrogen-mimicking chemicals [37]. DEHP (300 mg/kg/day) exposure for 16 days increased intestinal GUS enzyme activity and circulating estradiol levels, whereas 32 days of DEHP exposure reduced intestinal GUS activity and circulating estradiol levels compared to controls in mice [37]. Further, DEHP (300 mg/kg/day) induced some changes in the bacterial community that were reflected in the GUS and estradiol levels [37].

Interestingly, some specific bacterial species have been associated with reproductive aging. A study on gut microbial diversity in reproductive-aged versus menopausal women found 90 differential bacterial taxa between pre- and post-menopausal women [16]. *Roseburia* is the predominate taxon in the gut that discriminated between pre- and post-menopausal women, with higher *Roseburia* abundance observed in pre-menopausal women compared to post-menopausal women [16]. Interestingly, DEHP (500 mg/kg/day) significantly increased gut *Roseburia* in mice [41]. Additionally, gut *Firmicutes* abundance was low, and the gut *Bacteroidetes* population was enriched in post-menopausal women and POI patients compared to controls [16]. The *Firmicutes/Bacteroidetes* (*f/b*) ratio has been suggested as a hallmark of reduced estrogen levels observed in reproductively senescent rats [41]. One study suggests that the DEHP-induced increase in the *f/b* ratio may be responsible for DEHP-induced female reproductive toxicity, whereas another study demonstrated that DEHP (300 mg/kg/day) exposure at a lower dose has the opposite effect, decreasing the *f/b* ratio which may contribute to reduced estrogen levels, accelerating ovarian aging [37, 41].

Another hypothesis of researchers studying the effects of phthalates on reproductive dysfunction influenced by the gut microbiota suggests that gut microbial dysbiosis may increase intestinal permeability and alter levels of gut metabolites, allowing LPS, a known endotoxin, to be transported to the ovary [16]. High-dose DEHP exposure for 30 days significantly altered fecal microbiota and fecal metabolite levels (500-1500 mg/kg/day) and increased LPS levels in mouse ovaries and sera (1000-1500 mg/kg/day) compared to controls [30, 41]. LPS altered ovarian physiology in a similar manner to in vivo DEHP exposure and in vitro MEHP exposure [30, 42]. Due to this relationship, Xu et al. investigated the effects of MEHP and LPS co-exposure on cultured KGN and primary mouse granulosa cells. They found that MEHP synergized with LPS to activate the NF-κB signaling pathway and induce inflammatory apoptosis of granulosa cells, which may play a role in phthalate-induced accelerated ovarian aging if this relationship holds in vivo [30].

Fecal microbiota transplant (FMT) from young mice to aged mice significantly reduced follicular atresia and apoptosis, improving their fertility [16]. Interestingly, another study found that FMT from young zebrafish to aged zebrafish combated the effects of perfluorobutanesulfonate exposure, promoting oogenesis and reducing the rate of perfluorobutanesulfonate-induced malformations in the offspring of exposed fish [16]. These studies, combined with the evidence that DEHP-induced ovarian aging is mediated in part by the DEHP-induced disruption in the intestinal microbiota, suggest that FMT may be useful as a therapy for phthalate-induced infertility and perhaps, even reverse some of the age-related effects seen in ovaries of phthalate exposed organisms.

## Conclusions

Phthalate exposure plays a complicated role in accelerating ovarian aging. Phthalates alter steroidogenesis and folliculogenesis through numerous mechanisms, ultimately dysregulating estrous cyclicity and decreasing fertility in a manner consistent with reproductive aging [2, 4, 38]. Phthalates and their metabolites can alter reproductive endpoints and ovarian processes directly or through signaling from other organs such as the brain and gut [2, 4, 37, 41]. The brain and pituitary regulate ovarian aging through FSH and LH signaling, which are dysregulated with phthalate exposure [2, 4, 15]. Additionally, phthalate exposure triggers release of LPS into the bloodstream that causes subsequent adverse effects in the ovary [30]. Interestingly, alterations in the gut microbiome can directly alter circulating estrogen levels, a hallmark of ovarian aging [16]. Phthalate exposure alters the ovarian microenvironment by increasing inflammation and oxidative stress, which contributes to accelerated ovarian aging in numerous ways [1, 25, 29, 32, 39, 51]. Phthalate exposure also increases ovarian autophagy, mitochondrial dysfunction, and apoptosis, which ultimately increase follicular atresia and accelerate depletion of the follicle reserve, a primary phenotype of ovarian aging [2, 4, 25–32]. Considerable evidence exists that phthalates accelerate ovarian aging, and phthalate-induced accelerated ovarian aging occurs through a variety of interlinked mechanisms.

Growing evidence of DEHP-induced toxicity has moved some industries to replace DEHP with other phthalates such as DiNP and DEHTP [4, 43]. A growing body of research exists on the effects of DiNP on ovarian aging, but little research exists on the effects of non-DEHP/DiNP phthalates or phthalate mixtures on ovarian aging despite their environmental relevance. Further, humans are exposed to a mixture of phthalates daily [2]. Thus, it is critical to investigate the effects of non-DEHP/DiNP phthalates and environmentally relevant phthalate mixtures on ovarian aging. Studies on mixtures are especially important, as phthalates can have opposite effects to one another that may neutralize each other, or they may work synergistically to produce a more pronounced effect than they do individually [92].

Many studies investigated the effects of high-dose phthalates on reproductive and ovarian endpoints. These studies are important to elucidate mechanisms; however, phthalates are endocrine-disrupting chemicals that often exhibit non-monotonic dose responses, so the phthalates may exhibit larger effects at environmentally relevant doses than at high doses [93]. Thus, it is important to also study the effects of environmentally relevant doses of phthalates on reproductive and ovarian endpoints.

Finally, many studies reviewed in this article investigated the effects of acute or short-term phthalate exposures on reproductive endpoints and ovarian aging; however, humans are chronically exposed to phthalates throughout their lives, so it is also important to investigate the effects of chronic long-term phthalate exposures on the ovary.

##  Key References

- Balough JL, Dipali SS, Velez K, Kumar TR, Duncan FE. Hallmarks of female reproductive aging in physiologic aging mice. Nat Aging. 2024 Dec 13;4(12):1711–30.
  - ◌ This paper reviewed the hallmarks of ovarian aging in mice.
- Fletcher E, Santacruz-Márquez R, Mourikes V, Neff A, Laws M, Flaws J. Effects of Phthalate Mixtures on Ovarian Folliculogenesis and Steroidogenesis. Toxics. 2022 May 16;10(5):251.
  - ◌ This review examined the role of exposure to phthalate mixtures on ovarian folliculogenesis and steroidogenesis.
- Yang L, Chen Y, Liu Y, Xing Y, Miao C, Zhao Y, et al. The Role of Oxidative Stress and Natural Antioxidants in Ovarian Aging. Front Pharmacol. 2021 Jan 14;11:617843.
  - ◌ This review elucidated the role of oxidative stress in ovarian aging.
- Zhu X, Li H, Xue T, Wang S, Zhu R, Luo J, et al. Mechanistic study on the role of multi-pathway autophagy in ovarian aging: literature review. Apoptosis [Internet]. 2025 Sep 16 [cited 2025 Oct 12]; Available from: https://link.springer.com/10.1007/s10495-025-02181-2
  - ◌ This paper explored the molecular mechanisms of autophagy and their role in ovarian aging.
- Mara JN, Zhou LT, Larmore M, Johnson B, Ayiku R, Amargant F, et al. Ovulation and ovarian wound healing are impaired with advanced reproductive age. Aging. 2020 May 14;12(10):9686–713.
  - ◌ This paper established that advanced reproductive age is associated with dysregulation of ECM remodeling.
- Huang F, Cao Y, Liang J, Tang R, Wu S, Zhang P, et al. The influence of the gut microbiome on ovarian aging. Gut Microbes. 2024 Dec 31;16(1):2295394.
  - ◌ This review elucidated the influence of the gut microbiome on ovarian aging.

## Data Availability

No datasets were generated or analysed during the current study.
